# Branched-chain amino acid depletion conditions bone marrow for hematopoietic stem cell transplantation avoiding amino acid imbalance-associated toxicity

**DOI:** 10.1016/j.exphem.2018.04.004

**Published:** 2018-07

**Authors:** Adam C. Wilkinson, Maiko Morita, Hiromitsu Nakauchia, Satoshi Yamazaki

**Affiliations:** a Institute for Stem Cell Biology and Regenerative Medicine, Stanford University School of Medicine, Stanford, CA, USA; b Department of Genetics, Stanford University, Stanford, CA, USA; c Department of Haematology, University of Cambridge, Cambridge, UK; d Division of Stem Cell Therapy, Center for Stem Cell Biology and Regeneration Medicine, Institute of Medical Science, University of Tokyo, Tokyo 108-8639, Japan

## Abstract

Hematopoietic stem cells (HSCs) are used clinically in bone marrow (BM) transplantation due to their unique ability to reform the entire hematopoietic system. Recently, we reported that HSCs are highly sensitive to valine, one of the three branched-chain amino acids (BCAAs) in addition to isoleucine and leucine. Dietary depletion of valine could even be used as a conditioning regimen for HSC transplantation. Here, we report that HSCs are highly sensitive to the balance of BCAAs, with both proliferation and survival reduced by BCAA imbalance. However, low but balanced BCAA levels failed to rescue HSC maintenance. Importantly, in vivo depletion of all three BCAAs was significantly less toxic than depletion of valine only. We demonstrate that BCAA depletion can replace valine depletion as a safer alternative to BM conditioning. In summary, by determining HSC metabolic requirements, we can improve metabolic approaches to BM conditioning.

## Highlights

• Branched-chain amino acid (BCAA) imbalance (low valine and high isoleucine/leucine) inhibits hematopoietic stem cell (HSC) proliferation and survival.
• Low BCAA culture does not block HSC growth but poorly supports HSC maintenance.
• Dietary BCAA depletion conditions the mouse bone marrow (BM) for HSC transplantation.
• BCAA conditioning improves survival and red blood cell (RBC) counts compared with valine conditioning.

Bone marrow (BM) or hematopoietic stem cell (HSC) transplantation (HSCT) is a potentially curative treatment for a range of hematological disorders, such as immunodeficiency diseases [1]. However, “space” within the recipient's HSC niche 2, 3 must be first made to allow donor HSCs to engraft [4]. Unfortunately, the morbidity and mortality associated with traditional BM conditioning regimens, radiation and/or chemotherapy, currently limit the application of HSCT [5]. Recently, we reported that dietary valine depletion could be used to condition mice for HSCT [6]. Importantly, this type of metabolic BM conditioning was entirely reversible, with mice returning to full health and fertility after transplantation and return to a complete diet. Here, we describe important optimization of this novel conditioning approach for improved safety and tolerance based on further characterization of the metabolic sensitivity of HSCs.

Nearly 60years ago, Harper [7] proposed that amino acid imbalance could be a mechanism of disease. Valine is a one of three branched-chain amino acids (BCAAs) in addition to isoleucine and leucine. BCAA imbalance has been suggested to cause cellular toxicity, including neurotoxicity [8]. Stimulated by these reports, we investigated the sensitivity of HSCs to BCAA imbalance using ex vivo HSC expansion cultures. Expansion of mouse CD34^–^/loKit^+^Sca1^+^Lineage^–^ (CD34-KSL) HSCs [9] was determined after a 7-day culture in Dulbecco's modified Eagle's medium (DMEM)/F12-based self-renewal conditions [10]. DMEM/F12 medium was used because it contains approximately equimolar concentrations of BCAAs: 451 µmol/L valine, 415 µmol/L leucine, and 451 µmol/L isoleucine.

**Figure1:**
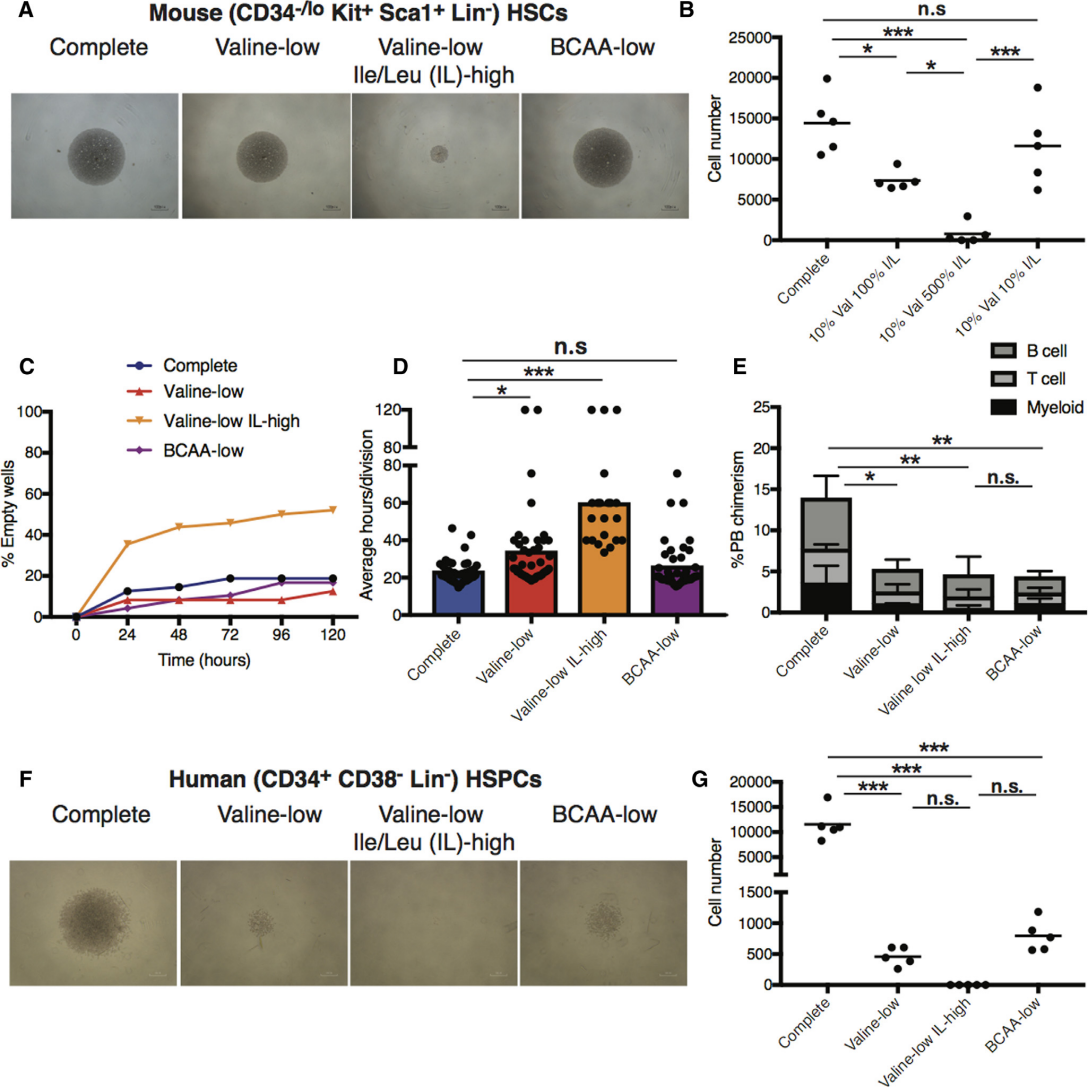
BCAA imbalance caused by low valine and high I/L blocks HSC expansion through reducing survival and inhibiting proliferation. **(A,B)** Mouse BM CD34-KSL HSCs were expanded (40 cells/well) for 7days in DMEM/F12-based media supplemented with 0.1% human serum albumin, stem cell factor (SCF, 50 ng/mL), thrombopoietin (TPO, 50 ng/mL), and 1% S-clone SF-O3 medium supplement. Representative colony images (4 × magnification) are shown in **(A)** and average cell numbers per well in **(B). (C,D)** Single HSCs were monitored over 5days in the media described above. The percentage of empty wells in shown in **(C)**. Estimated average number of hours per cell division event based on total number of cells at day 5 in **(D)**. Forty-eight cells were analyzed per condition. **(E)** Average donor PB chimerism ± SEM from competitive transplantation assays using 7-day cultured HSCs from **(A)** at 16 weeks after transplantation. C57BL/6 Ly5.1 HSCs were injected into irradiated C57BL/6 Ly5.2 mice (5 mice/condition) alongside 106 Ly5.1/Ly5.2 whole BM competitor cells, as described previously [10]. All animal experiments described herein followed guidance and approval from the Animal Care and Use Committee, Institute of Medical Science, University of Tokyo, or the Administrative Panel on Laboratory Animal Care, Stanford University. **(F,G)** Human CD34^+^CD38^–^Lineage^–^ HSPCs from umbilical cord blood (kindly provided the Stanford Binns Cord Blood Program) were expanded (300 cells/well) for 7days in DMEM/F12-based media supplemented with 1% bovine serum albumin, SCF (50 ng/mL), TPO (50 ng/mL), FLT3L (50 ng/mL), interleukin-6 (IL-6, 20 ng/mL), IL-3 (20ng/mL), and 1% S-clone SF-O3 medium supplement, as described previously [6]. Representative colony images are shown in **(F)** and average cell numbers per well ± SEM (*n* = 5) are shown in **(G)**. Statistically significant differences (one-way ANOVA) are denoted by asterisks: *p > 0.05, **p > 0.01, ***p > 0.001. n.s.=not significant.

**Supplementary Figure E1:**
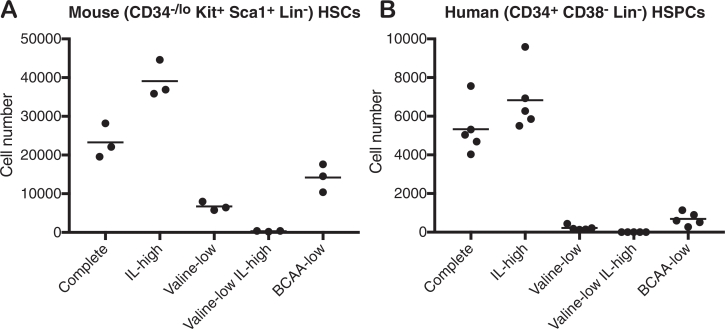
(**A**) Mouse BM CD34^−^KSL HSCs were expanded (40 cell/well) for seven days in DMEM/F12-based medias supplemented with 0.1% human serum albumin, SCF (50ng/mL), TPO (50ng/mL), and 1% S-clone SF-O3 medium supplement. (**B**) Human CD34^+^CD38^−^Lineage^−^ HSPCs from umbilical cord blood were expanded (300 cells/well) for seven days in DMEM/F12-based medias supplemented with 1% BSA, SCF (50ng/mL), TPO (50ng/mL), FLT3L (50ng/mL) IL-6 (20ng/ml), IL-3 (20ng/ml), and 1% S-clone SF-O3 medium supplement.

When the concentration of valine was reduced to ∼10% in this context (valine-low), we only mildly inhibited HSCexpansion (Figures 1A and 1B). However, when valine was reduced to ∼10% while the concentration of isoleucine/leucine (I/L) was simultaneously increased fivefold (valine-low, I/L-high), HSC expansion was blocked completely (Figures 1A and 1B). These results reproduced our initial screening [6], which used medium containing a similar BCAA imbalance. In contrast, increasing I/L concentrations fivefold in complete conditions (I/L-high) increased HSC expansion (Supplementary Figure E1, online only, available at www.exphem.org). Notably, reducing all three BCAAs to ∼10% in BCAA-low conditions (Figures 1A and 1B) only resulted in a modest, nonstatistically significant reduction in HSC expansion. Through single-cell assays, we found that BCAA imbalance (valine-low, I/L-high) blocked HSC expansion through a combination of increasing cell death and inhibiting proliferation (Figures 1C and 1D). However, whereas BCAA-low conditions did not influence HSC proliferation or survival significantly, it was not able to sustain in vivo function of HSCs activity, as indicated by decreased reconstitution capability of these cultured HSCs (Figure 1E). We therefore conclude that BCAA imbalance reduces HSC proliferation and survival, whereas low valine results in poor HSC maintenance.

To further investigate the translational potential of these findings, we investigated the consequences of BCAA imbalance on human cord blood-derived CD34^+^CD38^–^Lineage^–^ hematopoietic stem and progenitor cells (HPSCs). As seen with mouse HSCs, BCAA imbalance (valine-low, I/L-high) caused greater toxicity, with essentially no cells surviving the 7-day culture (Figures 1F and 1G). However, whereas BCAA-low conditions rescued mouse HSC expansion, it failed to rescue human HSPC expansion (Figures 1F and 1G). This is likely due to our previous finding that human HSPCs are also sensitive to leucine depletion [6].

**Figure2:**
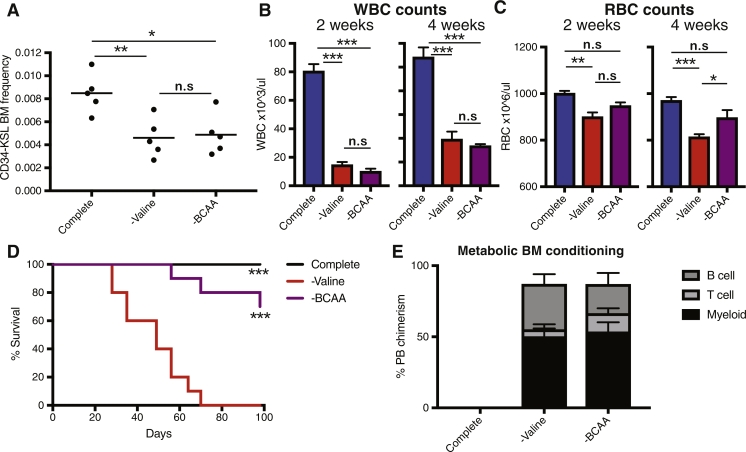
Dietary BCAA depletion is a less toxic metabolic conditioning regimen for HSCT. **(A–C)** C57BL/6 mice were fed a complete, –valine, or –BCAA diet (Research Diet, Inc.). Average BM and PB parameters were determined (*n* = 5 mice/condition). **(A)** BM CD34-KSL frequency after 4 weeks. **(B)** PB whole blood cell counts after 2 and 4 weeks. **(C)** PB red blood cell counts after 2 and 4 weeks. Statistically significant differences (one-way ANOVA) are denoted by asterisks: *p > 0.05, **p > 0.01, ***p > 0.001. n.s.=not significant. **(D)** Survival of C57BL/6 mice on complete, –valine, and –BCAA diets (10 mice/condition). Statistical testing was done using the log–rank test (***p > 0.001). **(E)** NOG mice were fed complete, –valine, or –BCAA diets for 2 weeks after an initial 48-hour fast and then injected with 104 (C57BL/6) BM KSL cells and the complete diet was restored gradually. No mortality was observed during transplantation. Average donor PB chimerism ± SEM at 16 weeks after transplantation (*n* = 4–5 mice per condition) is shown.

One concern with our initial proof-of-concept demonstration of metabolic BM conditioning [6] was the potential toxicity of dietary valine depletion. Although the effects of this metabolic conditioning approach were entirely reversible in mice after treatment (unlike radiation conditioning), the conditioning process did cause on-treatment side effects. Given that the imbalance of amino acids (and particularly BCAAs) is associated with systemic toxicities [8], we wondered whether our new understanding that HSCs were dependent of valine irrespective of BCAA balance could be used to improve non-chemoirradiative HSCT. We initially compared the consequences of dietary valine and BCAA depletion on hematopoiesis in mice. Consistent with our in vitro data, a BCAA-free (–BCAA) diet largely mirror a valine-free (–valine) diet, displaying reduced phenotypic HSC frequencies within the BM (Figure 2A) and peripheral blood (PB) whole blood cell counts (Figure 2B).

Interestingly, although we observed a reduction in red cell blood counts after a –valine diet, this anemia was milder with a –BCAA diet (Figure 2C). This suggests erythropoiesis may also be sensitive to BCAA imbalance and implies that the –BCAA diet is better tolerated. Our previous –valine dieting experiments had also been hampered by mortality after 4 weeks [6]. We therefore investigated the survival of mice on –valine and –BCAA diets directly (Figure 2D). Mice survived for a median of 49days on the –valine diet. In contrast, a –BCAA diet was much better tolerated by mice, with all mice surviving >7 weeks and 70% surviving 14 weeks. These data are also consistent with the report that a –valine diet caused neurotoxicity in rats, but this was not observed after a –BCAA diet [8]. Combined, these data suggest that the use of a –BCAA diet displays better safety and tolerance compared with a –valine diet.

Finally, we wanted to confirm that a –BCAA diet could be used as efficiently as a –valine diet to condition the BM for HSCT. We conditioned NOD.Cg-Prkdcscid Il2rgtm1Sug/ShiJic (NOG) mice [11] for 2 weeks and then transplanted 104C57BL/6 (C57BL/6NCrSlc) KSL cells before returning the diet to normal. Both –BCAA and –valine diets resulted in close to 100% PB chimerism in the long term (Figure 2E). A –BCAA diet therefore affords comparable BM conditioning for HSCT, but with reduced nonspecific toxicity compared with a –valine diet.

In summary, we have demonstrated that BCAA balance can resolve the sensitivity of HSCs to valine via its influence on cell cycle progression, survival, and influence on HSC maintenance. From these insights, we have developed a second-generation metabolic BM-conditioning regimen using BCAA depletion, which displays reduced in vivo systemic toxicity compared with valine depletion. These data provide an important step toward optimizing safe and well-tolerated metabolic BM-conditioning approaches for HSCT.

We hope this further understanding of the metabolic requirements of HSCs will lead to successful translation of safe metabolic BM-conditioning regimens to HSCT [12]. Metabolic BM conditioning may also be useful for basic HSC research, particularly for model animals in which radiation conditioning is challenging [13]. Through understanding the roles of BCAA-sensingmechanisms 14, 15, 16 and BCAA metabolism 17, 18 in HSC maintenance, we may also be able to develop pharmacological strategies for BM conditioning. Finally, it has been suggested recently that BCAA metabolism regulates leukemic progression 19, 20, which means that targeting BCAAs may also have therapeutic value in other contexts.
